# The Atlas of the Inferior Mesenteric Artery and Vein under Maximum-Intensity Projection and Three-Dimensional Reconstruction View

**DOI:** 10.3390/jcm13030879

**Published:** 2024-02-02

**Authors:** Hongwei Zhang, Shurong Liu, Bingqi Dong, Jing Liu, Xiaochao Guo, Guowei Chen, Yong Jiang, Yingchao Wu, Junling Zhang, Xin Wang

**Affiliations:** 1 Department of Gastrointestinal Surgery, Peking University First Hospital, Beijing 100034, China; 2 Department of Radiology, Peking University First Hospital, Beijing 100034, China

**Keywords:** inferior mesenteric artery, inferior mesenteric vein, anatomy, computed tomography

## Abstract

(1) **Background**: Understanding vascular patterns is crucial for minimizing bleeding and operating time in colorectal surgeries. This study aimed to develop an anatomical atlas of the inferior mesenteric artery (IMA) and vein (IMV). (2) **Methods**: A total of 521 patients with left-sided colorectal cancer were included. IMA and IMV patterns were identified using maximum-intensity projection (MIP) and three-dimensional (3D) reconstruction techniques. The accuracy of these techniques was assessed by comparing them with surgical videos. We compared the amount of bleeding and operating time for IMA ligation across different IMA types. (3) **Results**: Most patients (45.7%) were classified as type I IMA, followed by type II (20.7%), type III (22.6%), and type IV (3.5%). Newly identified type V and type VI patterns were found in 6.5% and 1% of patients, respectively. Of the IMVs, 49.9% drained into the superior mesenteric vein (SMV), 38.4% drained into the splenic vein (SPV), 9.4% drained into the SMV–SPV junction, and only 2.3% drained into the first jejunal vein (J1V). Above the root of the left colic artery (LCA), 13.1% of IMVs had no branches, 50.1% had one, 30.1% had two, and 6.7% had three or more branches. Two patients had two main IMV branches, and ten had IMVs at the edge of the mesocolon with small branches. At the IMA root, 37.2% of LCAs overlapped with the IMV, with 34.0% being lateral, 16.9% distal, 8.7% medial, and both the marginal type of IMV and the persistent descending mesocolon (PDM) type represented 1.4%. MIP had an accuracy of 98.43%, and 3D reconstruction had an accuracy of 100%. Blood loss and operating time were significantly higher in the complex group compared to the simple group for IMA ligation (*p* < 0.001). (4) **Conclusions**: A comprehensive anatomical atlas of the IMA and IMV was provided. Complex IMA patterns were associated with increased bleeding and operating time.

## 1. Introduction

Colorectal cancer is the third most common cancer and the second leading cause of cancer-related deaths [1]. Approximately 75% of colorectal cancers originate on the left side [2], encompassing the descending colon, sigmoid colon, and rectum. Laparoscopic surgery remains the primary treatment for left-sided colorectal cancer due to its minimally invasive nature. However, compared with traditional open surgery, laparoscopic procedures have certain disadvantages, such as limited surgical space and reduced tactile feedback, which may complicate vessel dissection, ligation, and hemostasis, making them more challenging. Therefore, a thorough preoperative understanding of the branching patterns of the vessels is essential for the success of laparoscopic surgery.

Typically, ligation of the inferior mesenteric artery (IMA) can be performed at two levels. The first, known as high ligation, involves ligating the root of the IMA. The second, known as low ligation, involves ligating the sigmoid artery (SA) and superior rectal artery (SRA) while preserving the left colic artery (LCA). The optimal level for IMA ligation is still a matter of debate [3,4,5]. Several studies suggested that the level at which the IMA was ligated did not significantly impact postoperative complications or survival outcomes [6,7]. Conversely, other research indicated that preserving the LCA provides better blood supply, reduces the likelihood of anastomotic leakage, and better preserves urinary and reproductive function [8,9,10]. Over the last few years, the anatomy of the inferior mesenteric vein (IMV) has also garnered increased attention, as selecting the appropriate ligation site of the IMV helps prevent postoperative congestion of the anastomosis [11,12,13]. Therefore, it is crucial to have a thorough understanding of the branching pattern of the IMV prior to surgery.

The CT-based maximum-intensity projection (MIP) technique has been widely utilized for vascular reconstruction. Three-dimensional (3D) reconstruction technology overcomes the limitations of two-dimensional imaging. Previous studies of IMA and IMV branching patterns have predominantly relied primarily on gross anatomy and autopsy [14,15], often with limited sample sizes. In this study, we retrospectively selected 521 patients with left-sided colorectal cancer and reviewed their contrast-enhanced CT images and laparoscopic videos. The branching patterns of the IMA and IMV were identified using MIP technology and/or 3D reconstruction. Additionally, we conducted a comparative analysis of blood loss and operating time associated with different types of IMA.

## 2. Materials and Methods

### 2.1. Patient Selection

**Figure 1:**
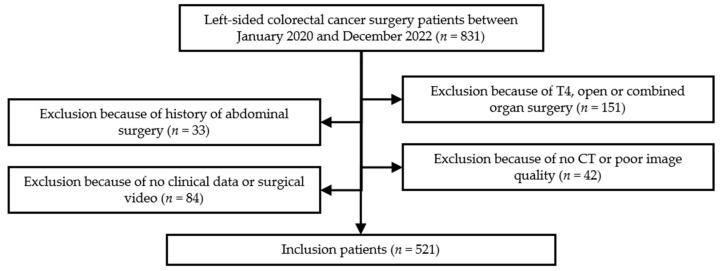
The flow diagram about the inclusion and exclusion criteria of patients.

We conducted a retrospective study on patients who underwent radical surgery for left-sided colorectal cancer at the Department of Gastrointestinal Surgery, Peking University First Hospital (PKUFH), from January 2020 to December 2022. The inclusion criteria were as follows: (1) radical surgery for left-sided colorectal cancer at our institution; (2) contrast-enhanced CT examination of the abdomen and pelvis at our institution, with all raw data of CT images accessible; (3) complete basic information, surgical records, and surgical video. The exclusion criteria included: (1) a history of abdominal surgery, including pancreatic, hepatobiliary, gastrointestinal, urinary, and gynecological surgeries; (2) poor quality of CT images, including motion artifacts, metal implant artifacts, and CT values less than 150 HU in the enhanced scan tube lumen; (3) severe arterial atherosclerosis and calcification leading to poor vascular imaging; (4) T4 staging, open surgery, or combined organ surgery patients; (5) patients with incomplete clinical data or surgical video. In total, 521 patients were included (Figure 1). This study was approved by the ethics committee of the PKUFH with the ethical approval number 2023-413-002.

### 2.2. Vascular Reconstruction Method

A Definition750 HD 64-slice spiral CT scanner was utilized for contrast-enhanced CT scanning. Two diagnostic teams, each comprising one surgeon and one radiologist, independently interpreted the vessel branching patterns using MIP technology and reconstructed thin-layer images with a thickness of 1.5 mm on the GEADW4.5 workstation. In cases where determining the origin of overlapping vessel structures was challenging, a senior radiologist physician determined the origin and name of the vessels from the original cross-sectional images and classified them according to the vessel branching pattern method. Additionally, all the raw data from the CT image were converted to the Digital Imaging and Communications in Medicine (DICOM) format. The data were then reconstructed to produce 3D digital vessel images using the Medical Imaging Three Divisional Visualization System (version 2.3.6.0, Shenzhen Xudong Digital Medical Imaging Technology Co., Ltd., Shenzhen, Guangdong, China).

### 2.3. Data Measurement

The horizontal distance from the root of the IMA to the LCA (D_LCA_), the horizontal distance from the root of the IMA to the IMV (D_IMV_), the 3D distance from the root of the IMA to the bifurcation of the iliac artery (D_IAB_), the 3D distance from the root of the IMA to the LCA (D_IMA_), and the 3D distance from the root of the LCA to the root of the SA (D_SA_) were measured. Blood loss and operating time during IMA ligation were estimated by reviewing the surgical video.

### 2.4. Types of IMA

Yada’s classification focuses on the LCA, while Zebrowski’s classification focuses more on the SA [16,17]. Inspired by these two classification methods, our study classified the IMA into six types based on the origin of the LCA as the primary classification condition and the number and location of SAs as the secondary classification condition. Type I refers to the common trunk of the SA and SRA, type II refers to the common trunk of the LCA and SA, type III refers to the LCA, SRA, and SA originating from the same point, type IV refers to the absence of an LCA, type V refers to one SA together with the SRA and the other SA together with the LCA, type VI refers to two separate LCAs from the IMA. Subtype ‘a’ refers to only one SA, subtype ‘b’, ‘c’, or ‘d’ refers to two SAs but from different parts of the LCA or SRA (Supplementary Figure S1).

### 2.5. Types of IMV

Regarding the location of IMV drainage, there are several variations: IMV drains into the superior mesenteric vein (SMV), IMV drains into the splenic vein (SPV), IMV drains into the confluence of the SPV and SMV, and IMV drains into the first jejunal vein (J1V). Our study classified the IMV into six types according to the number of branches of the main trunk of the IMV: No branch type, the main trunk of the IMV does not have the splenic flexure vein (SFV) and the left colic vein (LCV) branches; One branch type, the main trunk of the IMV has only one LCV branch, usually draining the transverse colon and splenic flexure; Two branches type, the trunk of the IMV has the SFV and LCV, draining the splenic flexure and descending colon, respectively; Three or more branches type, the main trunk of the IMV has three or more branches; Two IMVs type, there are two main IMV trunks in the descending colonic mesentery; IMV marginal type, the main trunk of the IMV is located at the edge of the mesentery and usually has many small branches (Supplementary Figure S2).

### 2.6. Intersection Patterns of LCA and IMV

According to the intersection patterns of the LCA and IMV at the level of the root of the IMA, there are six types of LCA/IMV intersection pattern, which we refer to as the PKUFH pattern: LCA medial type, LCA located medial to the IMV, i.e., D_LCA_ < D_IMV_; LCA overlapping type, LCA overlapping with the IMV, i.e., D_LCA_ = D_IMV_; LCA lateral type, LCA located lateral to the IMV, i.e., D_LCA_ > D_IMV_ and D_LCA_ − D_IMV_ ≤ 15 mm; LCA distal type, LCA located distal to the IMV, i.e., D_LCA_ > D_IMV_ and D_LCA_ − D_IMV_ > 15 mm; IMV marginal type, IMV located distal to the root of the IMA, i.e., D_IMV_ > 50 mm; Persistent descending mesocolon (PDM) type, the descending colon is located medially, and the sigmoid colon is located on the right side of the abdomen (Supplementary Figure S3).

### 2.7. Statistical Analysis

SPSS 29.0 software (IBM Corp., Armonk, NY, USA) was used for data analysis. Quantitative parameters in the study are presented as mean ± standard deviation. When data were not normally distributed, median and interquartile ranges were used instead. Student’s *t*-test was used to compare quantitative data between two groups, and the Mann–Whitney U test was used for skewed distributions. The chi-squared test was used for single-factor risk analysis. Differences were considered significant at *p* < 0.05.

## 3. Results

### 3.1. Patient Characteristics

A total of 521 patients were included in this study, comprising 306 males (58.7%) and 215 females (41.3%). The age range of the patients was from 28 to 89 years, with a median age of 63 years. The mean height was 166.46 cm, the mean weight was 67.12 kg, and the mean body mass index (BMI) was 24.16 kg/m^2^. Among the cases, there were 22 cases of left colon cancer, 132 cases of sigmoid colon cancer, and 367 cases of rectal cancer. Specifically, 308 patients underwent low anterior resection (LAR) surgery, 132 underwent radical surgery for sigmoid colon cancer, 32 underwent Miles surgery, 11 underwent Hartmann surgery, and 38 underwent left hemicolectomy, all of which were performed laparoscopically (Table 1).

### 3.2. IMA Types

**Figure 2:**
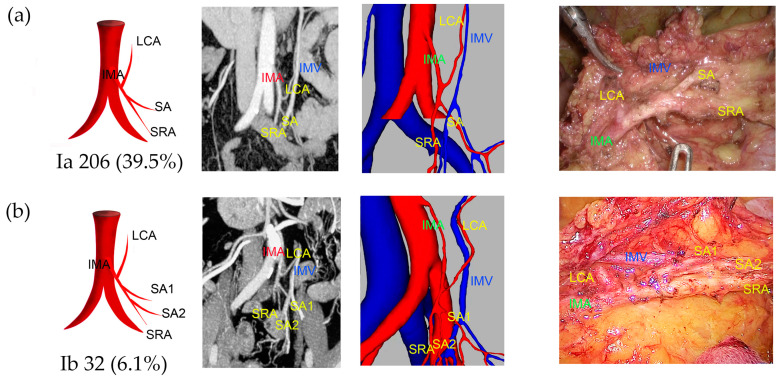
Representative images of IMA type I. The images are arranged according to the pattern image, MIP view, 3D reconstruction view, and laparoscopic view. (**a**) Type Ia: one SA and SRA originated from a common trunk. (**b**) Type Ib: two SAs originated from common trunk with SRA.

**Figure 3:**
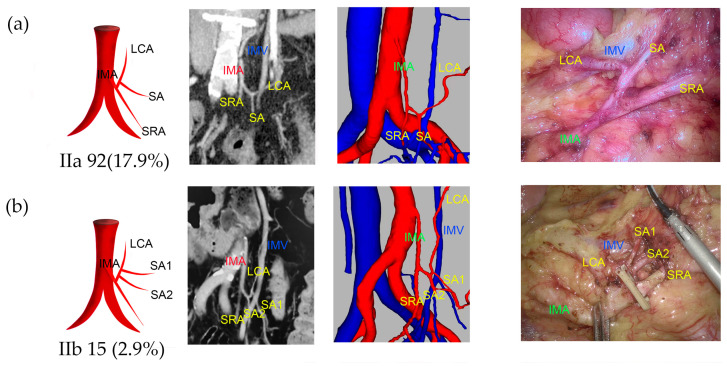
Representative images of IMA type II. The images are arranged according to the pattern image, MIP view, 3D reconstruction view, and laparoscopic view. (**a**) Type IIa: one SA and LCA originated from one common trunk. (**b**) Type IIb: two SAs originated from common trunk with LCA.

**Figure 4:**
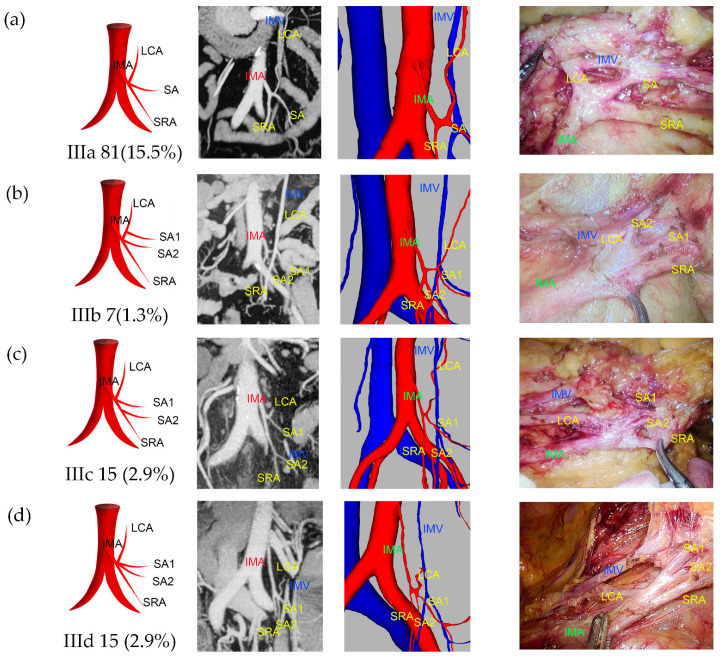
Representative images of IMA type III. The images are arranged according to the pattern image, MIP view, 3D reconstruction view, and laparoscopic view. (**a**) Type IIIa: LCA, SRA, and only one SA originated from the same point on IMA. (**b**) Type IIIb: LCA, SRA, and SA originated from the same point, with an additional SA which originated from LCA. (**c**) Type IIIc: LCA, SRA, and SA originated from the same point with an additional SA which originated from SRA. (**d**) Type IIId: LCA, SRA, and more than one SA which originated from the same point.

**Figure 5:**
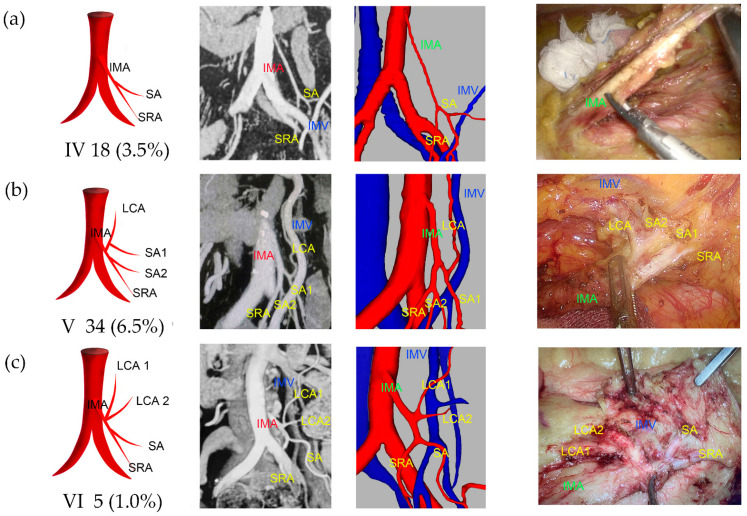
Representative images of IMA types IV to VI. The images are arranged according to the pattern image, MIP view, 3D reconstruction view, and laparoscopic view. (**a**) Type IV: LCA was absent. (**b**) Type V: one SA co-trunked with SRA, and the other SA co-trunked with LCA. (**c**) Type VI: two separate LCAs originated from the IMA.

Type I was observed in 238 patients (45.7%), of which 39.5% were classified as Ia and 6.1% as Ib (Figure 2). Type II was observed in 108 patients (20.7%), with 17.9% classified as IIa and 2.9% as IIb (Figure 3). Type III was observed in 118 patients (22.6%), with 15.5% classified as IIIa, 1.3% as IIIb, 2.9% as IIIc, and 2.9% as IIId (Figure 4). Type IV was observed in 18 patients (3.5%). Type V was observed in 34 patients (6.5%). Type VI was observed in five patients (Figure 5). The majority of IMAs (approximately 92.5%) originated from the third lumbar vertebral level, while approximately 4.2% and 3.3% originated from the second and fourth lumbar vertebral levels, respectively. The mean length of D_IAB_ was 45.42 ± 9.36 mm, D_LCA_ was 31.13 ± 17.12 mm, D_IMV_ was 23.57 ± 9.96 mm, D_IMA_ was 40.79 ± 10.77 mm, and the D_SA_ had an average length of 9.37 ± 8.27 mm (Supplementary Table S1).

### 3.3. IMV Types

**Figure 6:**
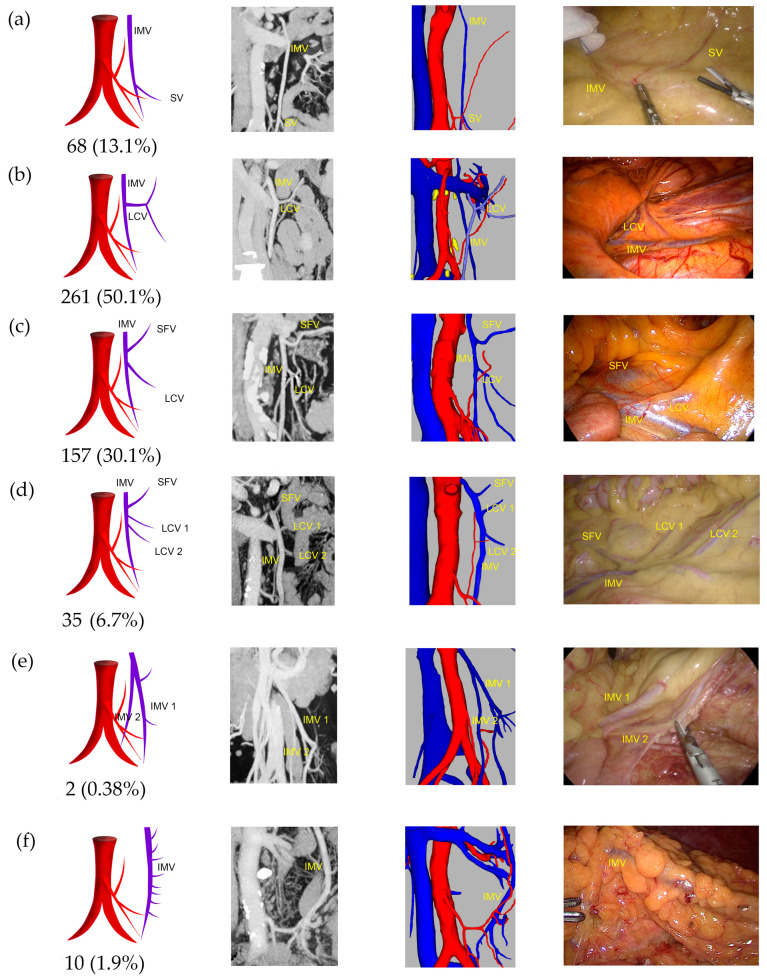
Representative images of IMV branching patterns. The images are arranged according to the pattern image, MIP view, 3D reconstruction view, and laparoscopic view. (**a**) No branch type: IMV main trunk had no SFV or LCV branch. (**b**) One branch type: IMV main trunk had only one LCV branch. (**c**) Two branches type: IMV main trunk had SFV and LCV branches. (**d**) Three or more branches type: IMV main trunk had three or more small branches. (**e**) Two IMVs type: two main IMV trunks. (**f**) IMV marginal type: the main trunk of IMV was located at the edge of the mesentery and had many small branches.

In 49.9% of patients, the IMV drained into the SMV, while 38.4% drained into the SPV, 9.4% into the confluence of the SPV and SMV, and 2.3% into the J1V (Supplementary Figure S4). Approximately 13.1% of IMVs had no branches above the level of the root horizon of the IMA, 50.1% had one branch, 30.1% had two branches, and 6.7% had three or more branches. Notably, two patients (0.38%) had two IMV trunks. Additionally, in another ten patients (1.92%), the main trunk of the IMV was located at the edge of the mesentery and had many small branches (Figure 6).

### 3.4. Intersectional Anatomical Patterns of LCA/IMV

**Figure 7:**
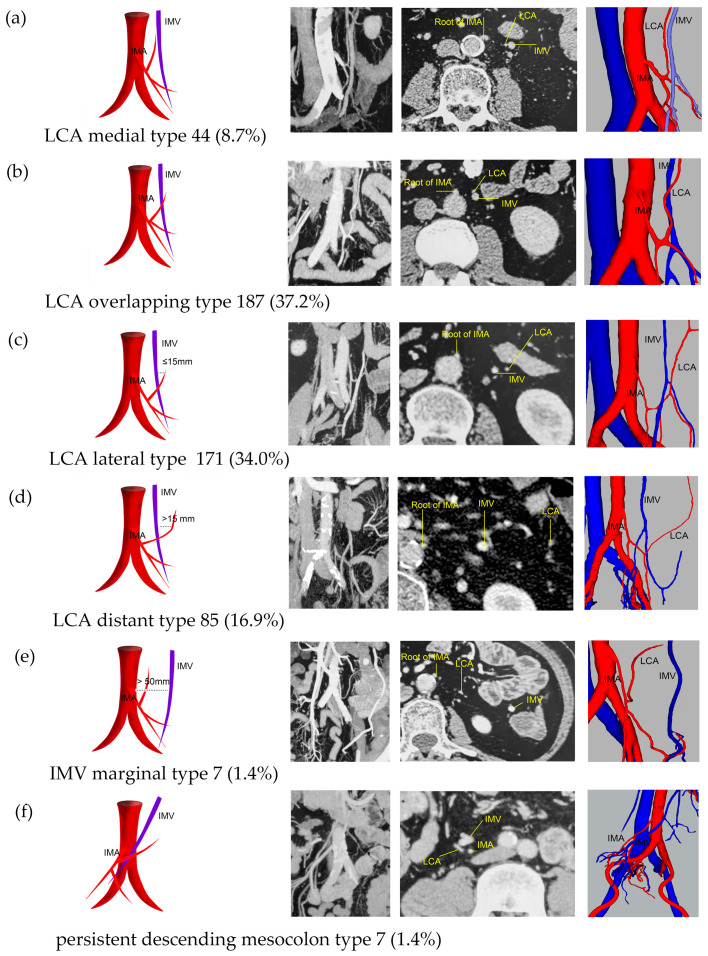
Representative images of LCA/IMV types. The images are arranged according to the pattern image, MIP view, transverse section view, and 3D reconstruction view. (**a**) LCA medial type: D_LCA_ < D_IMV_. (**b**) LCA overlapping type: D_LCA_ = D_IMV_. (**c**) LCA lateral type: D_LCA_ > D_IMV_ and D_LCA_ − D_IMV_ ≤ 15 mm. (**d**) LCA distant type: D_LCA_ > D_IMV_ and D_LCA_ − D_IMV_ > 15 mm. (**e**) IMV marginal type: D_IMV_ > 50 mm. (**f**) Persistent descending mesocolon (PDM) type: the descending colon was located medially, and the sigmoid colon was located on the right side of the abdomen.

The IMA was positioned anterior to the IMV in 370 (73.6%) patients and posterior to the IMV in 133 (26.4%) patients (Supplementary Figure S5). At the level of the root of the IMA, most LCAs overlapped with the IMV, accounting for 37.2%. This was followed by the LCA lateral type at 34.0%, LCA distal type at 16.9%, and LCA medial type at 8.7%. The IMV marginal type and PDM type each accounted for only 1.4% (Figure 7).

### 3.5. Accuracy of Vessel Reconstruction

The MIP and 3D visualization images were interpreted by two independent teams (T1, T2) for evaluation. Subsequently, the interpreted results of the vessel branching patterns were compared with the vessel patterns observed during surgery. The surgical procedure of high ligation of the IMA was excluded because of the difficulties in accurately classifying the IMA from surgical videos. When compared with the vessel patterns observed in the laparoscopic view, the overall accuracy of MIP and 3D visualization was 98.43% and 100%, respectively (Supplementary Table S2). Some of the MIP interpretations were not consistent with the actual situation because of vessels being compressed by metastatic lymph nodes, while others were related to excessive vessel density.

### 3.6. Blood Loss and Operating Time during IMA Low Ligation

Based on clinical experience, IMA types were categorized into simple and complex groups. The simple group consists of type I and type IV, while the complex group consists of type II, type III, type V, and type VI. Through a review of surgical videos with a preserved LCA, we compared the amount of blood loss and duration of the procedure during ligation of the IMA between the two groups in the LAR surgery or radical surgery for sigmoid colon cancer with low ligation of the IMA (*n* = 364). In comparison to the simple group, the complex group exhibited a higher amount of blood loss (9 mL vs. 6 mL, *p* < 0.001) and a longer operating time (25.44 min vs. 21.55 min, *p* < 0.001) (Supplementary Table S3).

## 4. Discussion

There is still ongoing debate regarding the optimal level of ligation of the IMA in surgery for rectal and sigmoid colon cancer. Several meta-analyses have indicated that there is no significant difference in postoperative complications and survival outcomes between high and low ligation approaches [18,19,20]. Conversely, other studies have highlighted the benefits of preserving the LCA, including providing better blood supply to the proximal anastomosis, reducing the likelihood of anastomotic leakage, and better urinary and reproductive function through better preservation of the superior hypogastric plexus [8,9,10]. Overall, based on some high-quality randomized controlled trials, low ligation has a clear advantage in preserving urinary and reproductive function, with no significant differences observed in terms of anastomotic leakage and survival outcomes [6,10]. Therefore, low ligation of the IMA appears to be the preferable choice. Similarly, controversy surrounds the method of ligation of the IMA in surgery for laparoscopic left hemicolectomy (LHC) and splenic flexure colectomy (SFC). Preservation of the IMA trunk may better maintain urinary and bowel function without increasing the incidence of postoperative complications [21,22]. In general, refined vascular ligation appears to be beneficial in surgeries for left-sided colorectal cancer.

The accurate preservation of blood vessels requires a high level of knowledge of vascular anatomy. There are several classifications for the IMA, among which the most classic are Latarjet’s classification, Zebrowski’s classification, and Yada’s classification. The Latarjet’s classification is based on whether the LCA and SA originate independently or in a fan-shaped manner. The Yada classification is based on the presence of the LCA, initial onset, and presence of common branches [16], whereas the Zebrowski classification is based on the number and distribution of SAs [17].

In recent years, several reviews have systematically reviewed the historical development of IMA classification and utilized meta-analysis to statistically evaluate the classical types [23,24,25]. The Yada and Zebrowski classifications seem to be the most widely applied; however, the Yada classification overlooks the description of the SA, and the Zebrowski classification is somewhat complex. Drawing upon our clinical experience and based on the Yada and Zebrowski classifications, we have classified the IMA into six types and several subtypes, each with a frequency of at least greater than 1%. IMA types I to IV are consistent with the new Yada classification modified by Murono et al. [26].

Our findings revealed that 45.6% of patients were classified as type I, while type II and type III patients accounted for 20.8% and 22.6%, respectively. Compared with a study of 468 patients, our study showed an increase in the proportion of type II patients and a decrease in the proportion of type III patients, while the proportion of type I patients was similar [26]. This difference may be due to differences in the study population. In particular, the D_SA_ in type II patients was significantly shorter than in type I patients. Therefore, misclassification of type II patients with a short D_SA_ as type III may be another possible reason. In our study, multiple SAs were observed in 25.3% of patients, which cannot be captured by the Yada classification, prompting us to introduce subtypes to offer valuable surgical insights while retaining Yada’s conciseness.

In recent years, venous drainage after colorectal cancer surgery has garnered increasing attention. To enhance venous drainage of the proximal anastomosis, Garcia Granero has proposed the “venous triangle theory”, which astutely selects the site for venous ligation to preserve more pathways for venous drainage [12]. Venous drainage in the distal anastomosis is common after LHC and SCF. Following ligation of the IMV trunk, venous blood flow in the distal colon can only drain through the pelvic veins, and, in some patients, poor venous return can lead to congestive ischemic colitis in the distal colon [11]. Some scholars advocate preserving the IMV trunk during surgery, and research has shown that preserving the IMV trunk may indeed improve postoperative outcomes [13]. 

In most cases, the IMV drains into the SPV, SMV, or the confluence of the SPV and SMV. Statistical data from various studies show significant variations [27]. In particular, our study found that, in 2.3% of patients, the IMV drained into the J1V. To the best of our knowledge, there does not seem to have been a specific classification method for the IMV in the past. In our study, we conducted a preliminary classification based on the number of IMV branches to provide a basis for personalized IMV ligation methods. According to our results, about half of the patients had only one branch, mainly draining the transverse colon and splenic flexure. Approximately 30% of patients had two branches, draining the splenic flexure and descending colon, respectively. In the future, as vein ligation methods evolve, we anticipate the development of more clinically relevant classification systems.

The IMV is typically ligated at the root of the IMA. When the LCA is preserved, the relative relationship between the LCA and the main trunk of the IMV is crucial for the surgical procedure. Patroni focused on the relationship between the LCA and IMV at the inferior margin of the pancreas. He categorized Latarjet’s classification into groups N and F, based on the distance between the LCA and IMV at the inferior margin of the pancreas [28]. In our study, we conducted a classification and statistical analysis of the anterior–posterior and left–right relationships between the LCA and IMV. The anterior–posterior relationship can assist the surgeon in determining the anatomical planes. In almost three quarters of cases, the IMA was found to be anterior to the IMV, consistent with previous research [26]. The left–right relationship can help predict the location of blood vessels. In approximately one third of cases, the LCA ran alongside the IMV, and, in a further one third of cases, the LCA was just lateral to the IMV. Preoperative understanding of the intersectional anatomical patterns of the LCA/IMV may reduce the possibility of inadvertent vessel injury.

A particular vascular pattern can be observed in the persistent descending mesocolon (PDM), known as PDM type. The PDM was first described by Morganstern in 1960 and is defined as an anomaly in which the descending colon is fixed to the mesentery, with the descending colon located medially and the sigmoid colon on the right side of the abdomen [29]. In our study, 1.4% of patients were found to have a PDM, which requires special attention. In most PDM cases, LCAs were far from the IMA root or absent. If surgeons are not aware of the anatomical variants of the colonic vessels before surgery, they may easily mistake the marginal vessels for the LCA and injure them during surgery, resulting in ischemia of the descending colon or even an unexpected colostomy [30].

Our research suggests that MIP technology is a cost-effective vascular reconstruction technique with acceptable accuracy. However, MIP may encounter difficulties in cases with complex vascular anatomy. When the vessel is highly curved and extends beyond the selected imaging thickness range, the vessel’s image may appear discontinuous or even missing. On the other hand, 3D reconstruction technology provides a rapid and accurate visual representation of the vessels. Its current use is limited due to its higher cost. In the majority of patients, the MIP technique can be used to quickly and accurately determine vessel classification. However, if the vessel branches are complicated or highly tortuous, the MIP technique may not accurately determine the classification, and, in such cases, three-dimensional reconstruction should be considered if low ligation is planned.

Our results revealed that the complex type group experienced significantly more blood loss and longer operating times compared to the simple type group. Similar findings have been reported in other articles [31]. There are two main reasons for this discrepancy. Firstly, intricate vessel branching patterns are more challenging and make the surgical procedure more demanding. Secondly, surgeons may lack a complete understanding of the vessel branching patterns prior to surgery. In laparoscopic surgery, the limited surgical space and reduced tactile feedback compound the procedural difficulty. Without a thorough understanding of the patient’s vascular classification before surgery, there is an increased risk of inadvertent vascular injury, leading to unnecessary bleeding. Therefore, accurate assessment of vascular patterns would be particularly beneficial in complex cases, especially for young surgeons with limited experience.

The study has limitations that need to be acknowledged. Firstly, this study was a single-center retrospective study, raising questions about its general applicability to other populations. To enhance the reliability of conclusions, subsequent research could include a multicenter prospective study. Secondly, owing to the high costs of 3D reconstruction, only 80 cases were reconstructed, which could have led to sampling bias. However, considering the high accuracy of MIP, the research results are not expected to significantly differ from real-world scenarios. Thirdly, in addition to intraoperative bleeding and operating time, it is important to consider the relationship between different vascular classifications and other complications, such as anastomotic leakage and mortality. Due to the many factors influencing anastomotic leakage and mortality, these were not considered in this study. Conducting a randomized controlled trial based on our vessel classification method will further demonstrate the clinical significance of vascular classification.

## 5. Conclusions

This study introduces a comprehensive and clinically relevant classification method for the patterns of IMA and IMV branching, as well as LCA/IMV intersectional patterns using MIP and 3D reconstruction. Additionally, we observed that complex IMA types were associated with increased intraoperative bleeding and prolonged operating time. Overall, this study contributes to our understanding of IMA and IMV branching patterns and their implications for surgical procedures.

## Figures and Tables

**Table 1:** General characteristics of patients.

Characteristics	Value
Number of patients	521
Age (years)	63.67 ± 11.54
Gender *n* (%)	
Male	306 (58.7%)
Female	215 (41.3%)
Height (cm)	166.46 ± 7.58
Weight (kg)	67.12 ± 11.05
BMI (kg/m^2^)	24.16 ± 3.20
Tumor location *n* (%)	
Rectum	367 (70.5%)
Sigmoid colon	132 (25.3%)
Descending colon	22 (4.2%)
Surgical method *n* (%)	
LAR	308 (59.1%)
Sigmoid colon surgery	132(25.3%)
Left hemicolectomy	38 (7.3%)
Miles	32 (6.2%)
Hartmann	11 (2.1%)
Ligation level *n* (%)	
Low	423 (81.2%)
High	98 (18.8%)

Continuous variables are expressed as the mean value ± SD. Categoric variables are expressed as numbers (percentages). Abbreviations: BMI, body mass index, LAR, low anterior resection.

## Data Availability

The data will be available on request.

## Supplementary Materials

The following supporting information can be downloaded at: https://www.mdpi.com/article/10.3390/jcm13030879/s1, Figure S1: IMA types; Figure S2: IMV types; Figure S3: Intersection patterns of LCA and IMV; Table S1: Measurement of IMA; Figure S4: Variable ways of IMV drainage; Figure S5: Representative images of IMV branching patterns; Table S2: The accuracy of MIP and 3D reconstruction; Table S3: Blood loss and operation time during ligation of IMA in LAR surgery or radical surgery for sigmoid colon cancer with low ligation of IMA.
