# Supplementary material for: The Atlas of the Inferior Mesenteric Artery and Vein under Maximum-Intensity Projection and Three-Dimensional Reconstruction View
Source: J Clin Med. 2024 Feb 2;13(3):879. doi: 10.3390/jcm13030879 (PMC10856009; doi:10.3390/jcm13030879)
Supplement: Supplementary file 1 [file jcm-13-00879-s001.zip › jcm-2773413-supplementary.pdf]

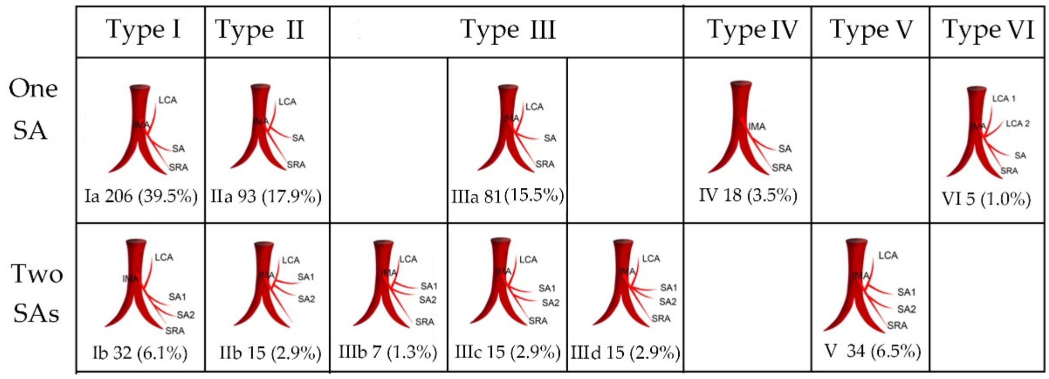

Figure S1. IMA Types. Type I refers to the common trunk of SA and SRA, type II refers to the common trunk of LCA and SA, type III refers to the LCA, SRA and SA originating from the same point, type IV refers to the absence of LCA. Type V refers to one SA together with the SRA and the other SA together with LCA. Type VI refers to two separate LCAs from IMA. Subtype 'a' refers to only one SA, subtype 'b', 'c' or 'd' refers to two SAs, but from different parts of LCA or SRA.

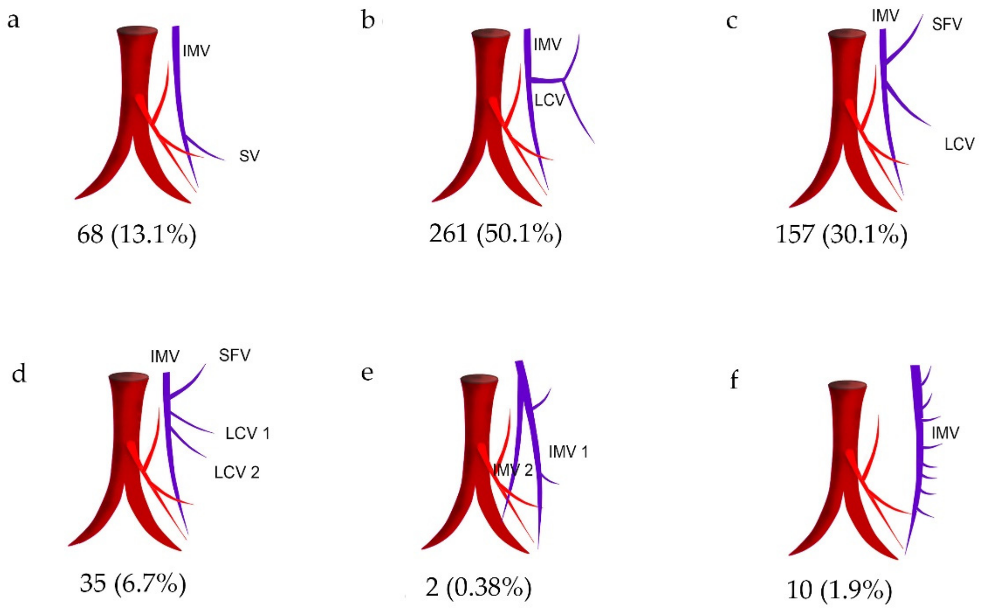

Figure S2. IMV Types. **(a)** No branch type: IMV main trunk had no SFV or LCV branch. **(b)** One branch type: IMV main trunk had only one LCV branch. **(c)** Two branches type: IMV main trunk had SFV and LCV branches. **(d)** Three or more branches type: IMV main trunk had three or more small branches. **(e)** Two IMVs type: two main IMV trunks. **(f)** IMV marginal type: the main trunk of IMV is located at the edge of the mesentery and has many small branches.

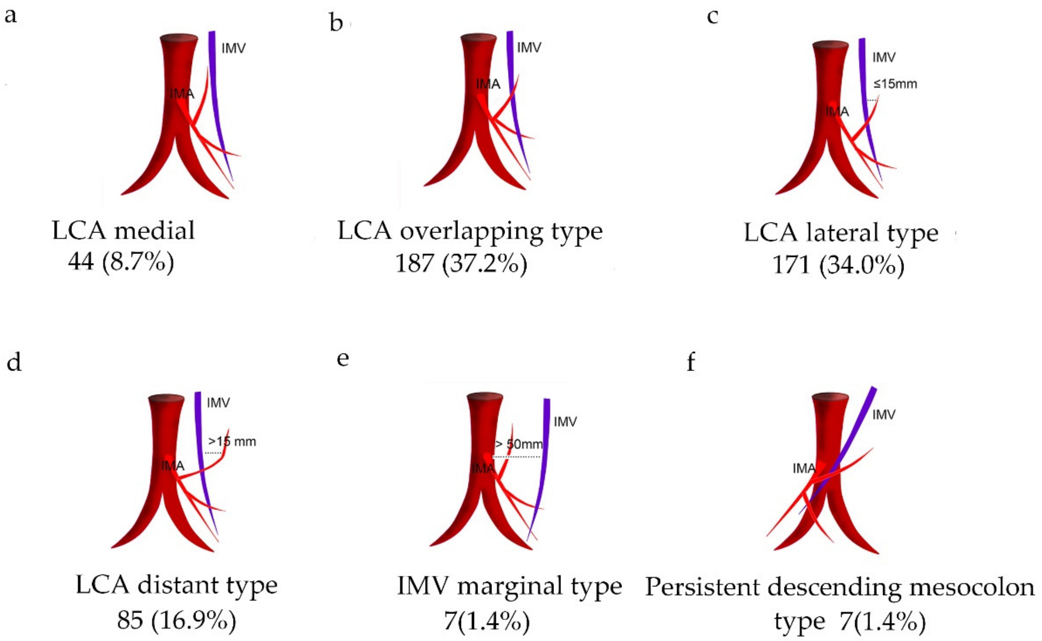

Figure S3. Intersection patterns of LCA and IMV. **(a)** LCA medial type: $D_{LCA} < D_{IMV}$. **(b)** LCA overlapping type: $D_{LCA} = D_{IMV}$. **(c)** LCA lateral type: $D_{LCA} > D_{IMV}$ and D_{LCA}- $D_{IMV} \leq$ 15mm. **(d)** LCA distant type: $D_{LCA} > D_{IMV}$ and D_{LCA}- D_{IMV} > 15mm. **(e)** IMV marginal type: D_{IMV} > 50mm. **(f)** Persistent descending mesocolon (PDM) type: the descending colon is located medially and the sigmoid colon is located on the right side of the abdomen.

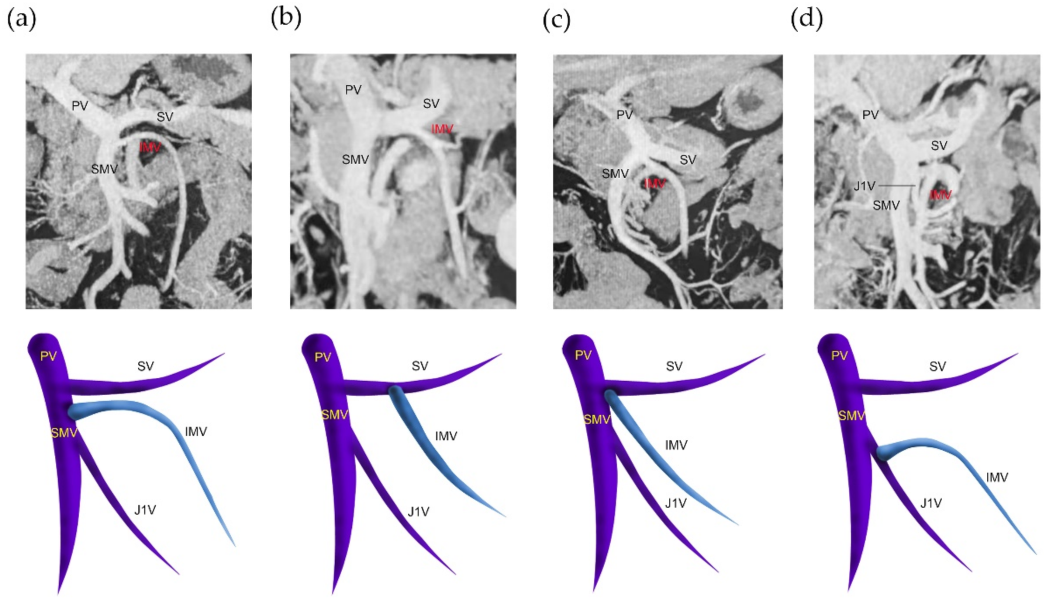

Figure S4. Variable ways of IMV drainage. The images arranged according to the MIP view and pattern image. **(a)** IMV drained into SMV. **(b)** IMV drained into SPV. **(c)** IMV drained into the confluence of SPV and SMV. **(d)** IMV drained into J1V.

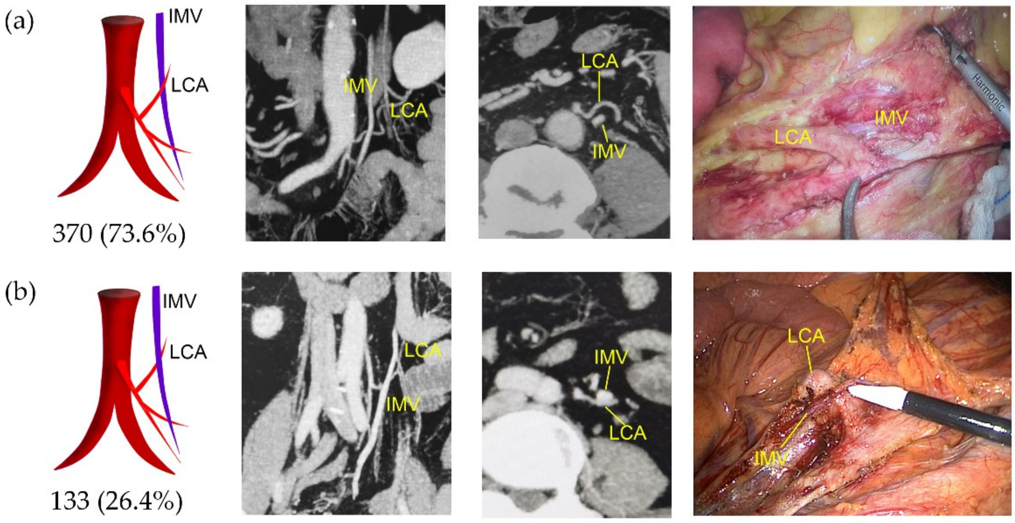

Figure S5. Representative images of LCA and IMV anteroposterior relation. The images arranged according to the pattern image, MIP view, transverse section view and laparoscopic view. **(a)** LCA lied anterior to IMV. **(b)** LCA lied posterior to IMV.

Table S1. Measurement of IMA.

Characteristics	Type I	Type II	Type III	Type IV	Type V	Type VI	p Value
D_{IAB} (mm)	46.93 ± 9.84	43.79 ± 8.41	44.26 ± 8.83	44.33 ± 9.24	44.94 ± 9.78	43.40 ± 8.56	0.036
D_{LCA}(mm)	29.07 ± 15.27	35.46 ± 21.81	32.15 ± 16.03	N/A	28.88 ± 14.91	27.00 ± 6.67	0.019
D_{IMV}(mm)	22.95 ± 9.29	23.01 ± 10.02	24.52 ± 9.24	31.22 ± 20.72	22.29 ± 7.13	24.00 ± 5.79	0.018
D_{IMA}(mm)	38.76 ± 9.70	43.94 ± 9.66	40.66 ± 12.53	53.61 ± 9.14	39.65 ± 9.16	34.40 ± 8.62	<0.001*
D_{SA}(mm)	14.29 ± 7.35	9.01 ± 6.61	N/A	N/A	8.26 ± 4.35	9.80 ± 4.82	<0.001*

Continuous variables were expressed as the mean value ± SD. N/As mean not applicable. *$p < 0.05$ (two-sided) was considered statistically significant. Abbreviations: IMA inferior mesenteric artery, D_{LCA} horizontal distance of IMA root to LCA, D_{IMV} horizontal distance of IMA root to IMV, D_{IAB} 3D distance of IMA root to the bifurcation of the iliac artery, D_{IMA} 3D distance of IMA root to LCA, D_{SA} 3D distance of LCA root to SA root.

Table S2 The accuracy of MIP and 3D reconstruction

		Team 1		Team 2		Average accuracy (%)	Total accuracy (%)
		Correct	Incorrect	Correct	Incorrect		
MIP	Sigmoid and rectal cancer (*n* = 423)	415	8	418	5	98.46	98.43
	Descending colon cancer (*n* = 22)	21	1	22	0	97.73	
3D reconstruction	Sigmoid and rectal cancer (*n* = 75)	75	0	75	0	100	100
	Descending colon cancer (*n* = 5)	5	0	5	0	100	

Abbreviations: MIP maximum intensity projection, 3D: three-dimensional

Table S3 Blood loss and operation time during ligation of IMA in LAR surgery or radical surgery for sigmoid colon cancer with low ligation of IMA

**Characteristics**	**Simple group (*n* = 160)**	**Complex group (*n* = 204)**	**p value**
Age (years)	64.72 ± 11.62	63.92 ± 11.61	0.513
Gender *n* (%)			
Male	96	123	1.000
Female	64	81	
Tumor diameter (cm)			
<4	65	74	0.460
≥4	95	130	
T staging *n* (%)			
T1-2	33	44	0.929
T3	127	160	
N staging *n* (%)			
N0	90	112	0.880
N1-2	70	92	
Tumor location (Distance from anus) (cm)			
<10	56	89	0.119
≥10	104	115	
Blood loss (ml)	6 (4, 8)	9(5, 13)	<0.001*
Operate time (min)	21.56 ± 4.53	25.43 ± 3.82	<0.001*

Continuous variables were expressed as mean value ± SD or the median (first quartile-third quartile). Categoric variables were expressed as numbers (percentages). *$p < 0.05$ (two-sided) was considered statistically significant. Abbreviations: IMA inferior mesenteric artery, LAR low anterior resection
